# Understanding public interest and needs in health policies through the application of social network analysis on a governmental Facebook fan page

**DOI:** 10.1186/s12889-020-09420-y

**Published:** 2020-09-07

**Authors:** Hsiang-Min Huang, Ching-Ju Chiu

**Affiliations:** 1 Institute of Public Health, College of Medicine, National Cheng Kung University, Tainan, Taiwan, Republic of China; 2 Institute of Gerontology, College of Medicine, National Cheng Kung University, No. 1, University Road, Tainan, 70101 Taiwan, Republic of China

**Keywords:** Health and welfare agencies, Facebook, Social network, Policy announcement, Public interest

## Abstract

**Background:**

This study analyzed the interactions between agencies, policies, and the interest of the public using a social network analysis.

**Methods:**

Open data on the 2017 Facebook fan page of the Ministry of Health and Welfare (MoHW) in Taiwan, including 18,193 messages, were analyzed by conducting a social network analysis, NodeXL (Network Overview, Discovery and Exploration for Excel), creating visualized explorations using size volumes to present the degree of strength between agencies and policies to further calculate the network centrality indicators of agencies and policies.

**Results:**

Agencies of the “Social and Family Affairs Administration” and “Health Promotion Administration” contributed the most policy posts. The policy of “Physical and mental health promotion” entailed the most agencies to be involved. The “Department of Nursing and Health Care” received the largest public response, for which “Long-term care” received the most public interest.

**Conclusions:**

A social network analysis of fan page of Taiwan’s top level health government agency can reveal the government’s most emphasized core policies, the strength of correlations between agencies and policies, and provide an understanding of public interest toward the policies.

## Background

National promotions related to health policies are often announced to the public through press releases, press conferences, or website announcements; however, public access to this information is mostly passive and limited, or portions of the information may be used to misconstrue policy by certain agents to mislead the public. However, with the continuous advancement of technology and the peak penetration rate of internet usage, the public can easily obtain complete information from social networks. Also, posts on social media are often accompanied by images, making them easy for the public to understand and increasing the view rate.

Related data pointed out that of social media networks, Facebook has the highest growth, nearly 80% of Taiwan’s population uses Facebook to collect or share information in 2016 [1]. Many for-profit organizations have grasped this trend and have adjusted their marketing and operating strategies, and Taiwan’s government departments have also broken out of the traditional mold and established various social media presences for social marketing to more effectively convey issues related to public health policy. The report pointed out that “Health 99”, the official website in Taiwan, used Facebook fan page which observed social media marketing not only allows the public to easily obtain information, but also optimizes efficiency [2] .

Taiwan’s top health government agency is the Ministry of Health and Welfare (MoHW). From the organizational structure chart of Taiwan’s MoHW [3], there are 8 business units (level-1 departments) and 6 subsidiary agencies (level-2 departments) primarily operating in the areas of medical insurance, social insurance, and public health issues. Furthermore, Taiwan’s MoHW regularly proposes annual plans to achieve their mission of national health and social benefits by requiring major departments to increase efficiency and avoid the waste of resources. The MoHW also recently provides real-time information on various policies on its Facebook fan page; however, after providing various types of health and welfare information, there has been little analysis as to the degree of interaction between the public and core policies.

This study proposes an analysis of social networks to explore the relationship between the primary promotional agencies of the MoHW and policy content on social media and the number of public replies in order to construct the relationship between MoHW policy promotion and public interest.

## Methods

### Data

In this study, the related MoHW departments are identified from the MoHW website and keywords for department and policy are defined based on the “MoHW 2017 Annual Projects Report” [4]. Public posts on social media in 2017 and the volume of replies are also collected. A total of 103 posts and 18,193 replies were collected.

### Measures

Agencies are defined based on the business departments and subordinate agencies on the MoHW administrative organization chart in Additional file 1 [3]. A total of 8 business departments were identified, those associated with social benefits are the Department of Nursing and Health Care, Social Assistance and Social Network, and Protective Services; those related to health services are the Department of Medical Affairs, Mental and Oral Health, Chinese Medicine and Pharmacy. The departments of Planning, and Social Insurance primarily tasked with the organization of joint policy and the consolidation of social insurance businesses.

A total of 6 subordinate agencies were identified, including the Centers for Disease Control (CDC), the Food and Drug Administration (FDA), the National Health Insurance Administration (NHA), the Health Promotion Administration (HPA), the Social and Family Affairs Administration (SFAA), and the National Pension Supervisory Committee (NPSC). They are separately responsible for acute infectious disease, food and drug safety, medical health insurance, chronic disease and promoting good health, minority groups and supporting families, and contract related departments. Aside from the 14 agencies listed here, a category for other agencies was added for a total of 15.

Policy comprises the 9 major goals of the MoHW 2017 Annual Project Report, and includes welfare services for minority groups, long-term care, protective services, medical care, prevention of infectious diseases and epidemics, food and drugs, physical and mental health promotion, and health insurance and pension budgeting. Aside from the 9 policies listed, a category for other policies was added for a total of 10.

Degree Centrality means that adjacent individual counts are used to measure the area centrality of networks, meaning the scope of individual control. If it is directional, they are further separated to out-degree centrality and in-degree centrality. Out-degree centrality shows the number of connections within an area so as to identify which agencies or policies have the most number of replies. In-degree centrality identifies individuals that pay higher attention due to their participation in replies. Betweenness Centrality refers to key bridges between the vertices within the network. In other words, higher betweenness centrality signifies key vertices since connections between vertices require passing through it. Closeness Centrality refers to measures of closeness between individuals and other individuals, with higher closeness centrality signifying that individuals are receiving related information at a faster rate.

### Statistical analysis

This study utilizes NodeXL (Network Overview, Discovery and Exploration for Excel) to conduct the social network-related analysis. A social network analysis is a sociological methodology that analyzes the interactions of connections and actors (individuals, organizations, nations, incidents) between various relationships to find the structure of social networks [5]. A social network relationship analysis can identify key roles and individuals who convey information between two or more groups, in which vertices can be labeled as one group and the degree of interaction between groups [6]. These results allow further presentation of the characteristics of overall social networks to determine the key points related to national policy announcements and the status of public reception.

Before the analysis, we must gain a further understanding of the key words that correspond to our explored departments and their policies to facilitate data organization for the future analysis. The keywords of each agency are determined by their public announcements on websites and their operational roles; the content of various policy keywords are determined using the MoHW 2017 Annual Project Plan. Next, NodeXL is utilized to collect the posts published on the MoHW fan page and the number of replies for analysis, creating visualized explorations using size volumes to present the degree of strength between agencies and policies to further calculate the network centrality indicators of agencies and policies. Indicators include degree centrality, betweenness centrality, and closeness centrality exploring core agencies and policies. Finally, public reply statuses are analyzed to understand the degree of participation of the public towards MoHW agencies or policies.

## Results

**Fig. 1:**
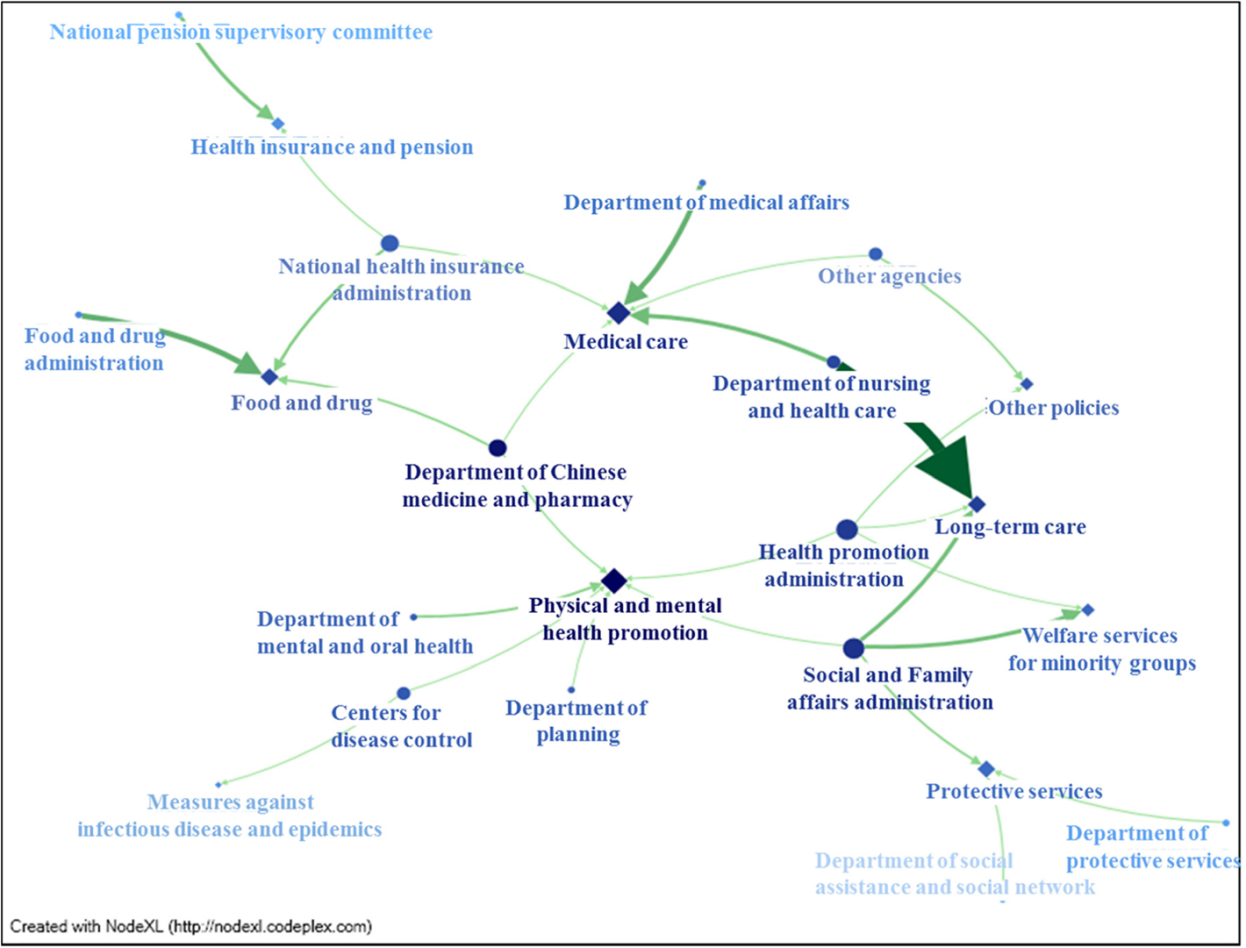
Network relationship diagram of all agencies (circled) and all policies (diamond) of the Ministry of Health and Welfare in Taiwan in 2017. Note: Deeper colored dots represent larger closeness centrality; larger dots represent larger degree centrality; thicker, deeper lines represent more replies

All public posts in 2017 were collected, for a total of 103 posts, from which the key words of agencies and policies were categorized and matched for a total of 27 types of posts containing related health and welfare information posted by agencies, with a total of 18,193 replies. Of the replies, posts related to nursing and health care combined with long-term care policy received the most replies (*n* = 5671) followed by food and drug policy posts issued by FDA (*n* = 2084), and the third being posts about medical care policy issued by the Department of Medical Affairs (*n* = 1847). Figure 1 shows lines of varying thicknesses representing the volume of replies.

### Network relationships between all agencies and policies

Figure 1 contains a total of 23 vertices (where one vertices represents an agency or policy) and 27 total edges, with a maximum geodesic distance (diameter) of 8, meaning that a maximum of 8 vertices connect two vertices, averaging 3.21 vertices. Also, a maximum degree centrality of 6 compared to Fig. 1 shows the maximum value to be “physical and mental health promotion,” signifying it as the policy with participation from the most agencies; within agencies, the maximum degree centrality is 4 and a comparison to Fig. 1 shows that “SFAA” and “HPA” agencies have the most policy execution with the largest sphere of influence, making them core agencies. Maximum betweenness centrality is 237.562, where when compared to Fig. 1, it is “physical and mental health promotion;” the maximum closeness centrality was 0.020, and when compared to Fig. 1, it is also “physical and mental health promotion.” In other words, physical and mental health promotion was the primary core policy at the time, and the public was able to quickly obtain related information.

**Fig. 2:**
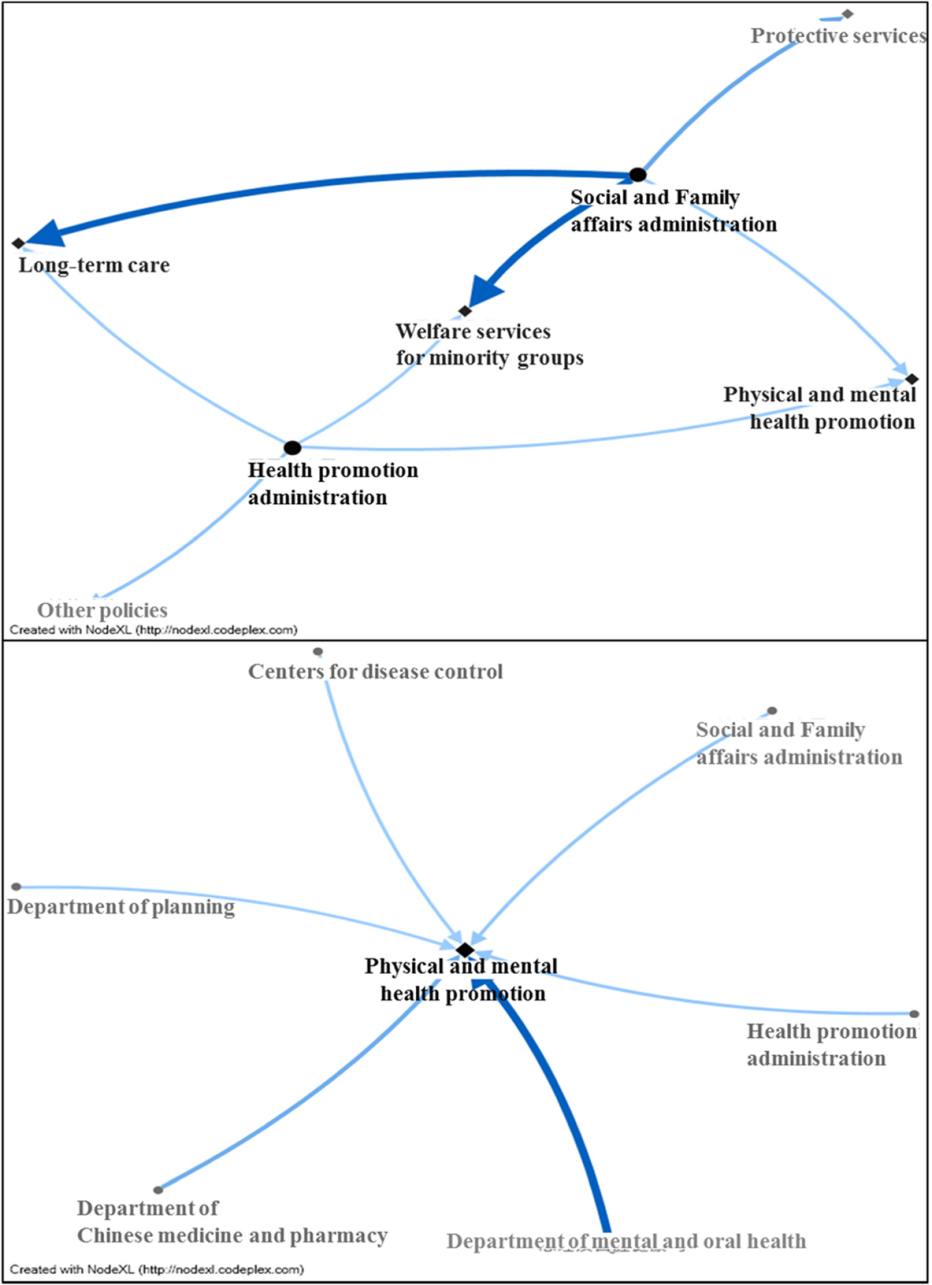
Core agencies and policies in 2017 Taiwan’s Ministry of Health and Welfare. Note: 1. The upper diagram presents core agencies (circled) and policy applications; the lower diagram presents core policies (diamond) and agency application. 2. Deeper colored dots represent larger closeness centrality; larger dots represent larger degree centrality; thicker, deeper lines represent more replies

Figure 2 shows core agencies (SFAA and HPA) and core policies (physical and mental health promotion) and their corresponding policies and agencies. Figure 2 shows that the core agencies promote policies related to long-term care, welfare services for minority groups, and physical and mental health promotion; SFAA promotes additional protective service-related policies, and HPA has other policies. Of these, SFAA’s welfare services for minority groups (*n* = 1326), long-term care (n-1295), and protective services (*n* = 443) ranked among the top 3 in terms of the degree of public response. Figure 2 also shows that core policies are being promoted by the Department of Mental and Oral Health (*n* = 592), the Department of Chinese Medicine and Pharmacy (*n* = 172), HPA (*n* = 39), SFAA (*n* = 37), CDC (n = 4), and the Department of Planning (n = 1), where the order above is ranked according to public response.

### Status of public response to agency postings

**Fig. 3:**
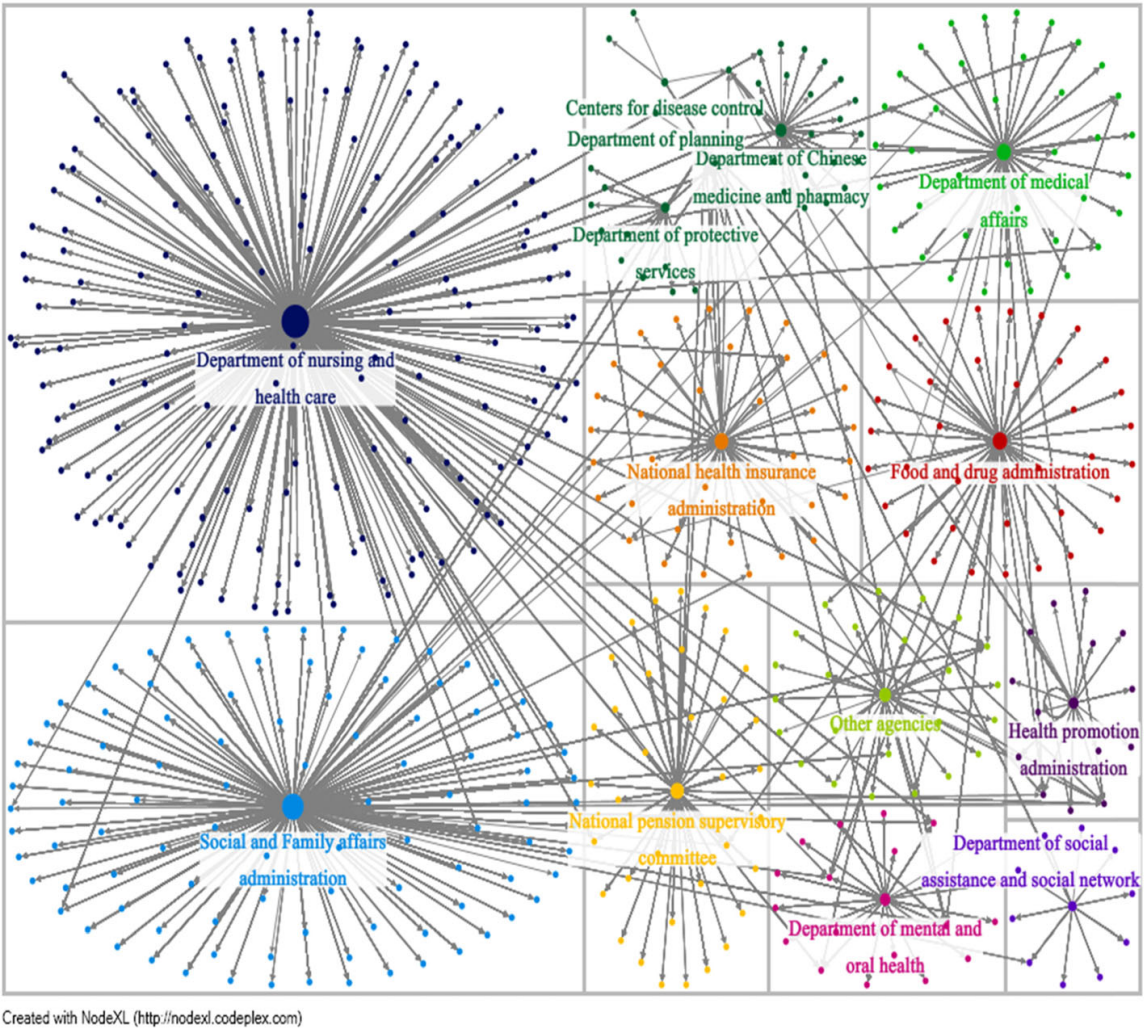
Associations among agencies of the Ministry of Health and Welfare and the density of the public responses. Note: Size of vertices indicate which agency receives the highest number of responses

Figure 3 is a visualization of the network relationship between agency posts and public response. There are known to be a total of 531 vertices with 14 vertices being agencies and the remaining being individuals, for a total of 18,193 edges. The maximum geodesic distance (diameter) is 4, meaning Fig. 3 has a maximum of 4 vertices connecting two vertices with an average of 3.50 vertices. Table 1 shows that out-degree centrality and maximum betweenness centrality have identical orders of agencies with the largest being the “Department of Nursing and Health Care” since it received the largest public response (out-degree: 194) and is a key communication pathway in the network (betweenness: 156100.104). The least active agency is the “Department of Planning” (out-degree:1; betweenness 0.000). The ranking of agencies is in the following order: SFAA, FDA, the Department of Medical Affairs, NHA, NPSC, other agencies, the Department of Chinese Medicine and Pharmacy, the Department of Mental and Oral Health, HPA, the Department of Protective Services, the Department of Social Assistance and Social Network, and CDC.

**Table 1:** Network centrality indicators of agencies and policies

**Agency**	**Out-Degree Centrality**	**Betweenness Centrality**
Department of nursing and health care	194	156,100.104
Social and Family affairs administration	118	96,446.879
Food and drug administration	47	38,693.049
Department of medical affairs	44	35,668.198
National health insurance administration	41	33,989.732
National pension supervisory committee	39	33,597.196
Other agencies	29	22,054.933
Department of Chinese medicine and pharmacy	25	20,403.471
Department of mental and oral health	21	16,926.273
Health promotion administration	17	11,438.557
Department of protective services	12	9556.125
Department of social assistance and social network	11	9550.283
Centers for disease control	5	2231.199
Department of planning	1	0.000
**Policy**	**Out-Degree Centrality**	**Betweenness Centrality**
Long-term care	193	156,021.169
Medical care	109	92,621.151
Food and drug	79	66,792.145
Protective services	52	45,829.367
Health insurance and pension	42	35,732.988
Physical and mental health promotion	41	33,734.765
Other policies	36	28,418.174
Welfare services for minority groups	29	20,799.983
Measures against infectious diseases and epidemics	4	1152.258

Note:

1. Out-Degree Centrality: shows the number of total edges within one area to show which agency or policy receives the highest number of responses

2. Betweenness Centrality: higher values indicate a key vertices, signifying it as an important bridge between different vertices within the network

### Status of public replies to agency postings

**Fig. 4:**
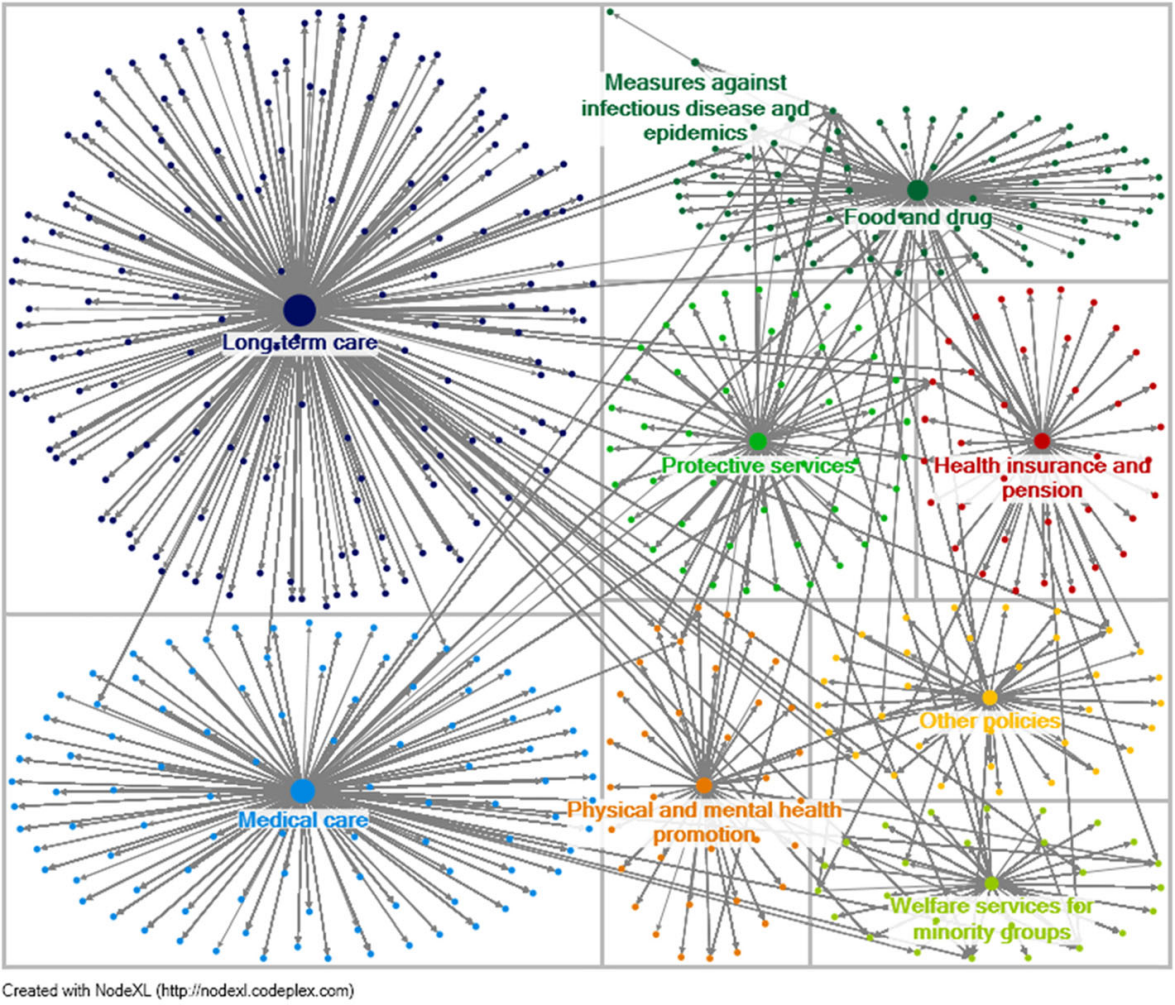
Associations among policies of the Ministry of Health and Welfare and the density of the public responses resulting. Note: Size of vertices indicate which policy receives the highest number of responses

Figure 4 is a visualization of the network relationship between agency posts and public replies. There are known to be a total of 527 vertices with 9 vertices being policy and the remaining being the individual members of the public, for a total of 18,193 edges. The maximum geodesic distance (diameter) is 4, meaning Fig. 4 has a maximum of 4 vertices connecting two vertices, for an average of 3.50 vertices. Table 1 shows that the order of out-degree centrality and maximum betweenness centrality have identical orders of policies with the highest being “long-term care” receiving the largest number of public replies (out-degree: 193) while also being a key communication pathway in the network (betweenness: 156021.169). The least active policy is “measures against infectious disease and epidemics” (out-degree: 4; betweenness: 1152.258). The ranking of the remaining policies is in the following order: medical care, food and drugs, protective services, health insurance and pensions, physical and mental health promotion, other policies, and welfare services for minority groups.

## Discussion

Nowadays, there were many social network research use NodeXL to explore the relationship between users [7–13]. Nevertheless, this is the first study exploring how government officials use the Facebook fan page to promote policies and analyze the interaction between agencies and people by using a social network analysis. We found that agencies of the “Social and Family Affairs Administration” and “Health Promotion Administration” contributed the most policy posts. The policy of “Physical and mental health promotion” entailed the most agencies to be involved. The “Department of Nursing and Health Care” received the largest public response, for which “Long-term care” received the most public interest.

With the continuous advancement of technology and the peak penetration rate of internet usage, the public can easily obtain complete information from social networks, and posts on social media are often accompanied by images, making them easy for the public to understand and increasing the view rate. In other words, the age of information is upon us and “internet media platforms” have become the bridge of communication between governments and the public. Social media has brought the changes in the communication landscape, and rapid increased globally, including in health care contexts [2–6, 14–16]. Most of the general public, and health-related professionals are using social media to communicate about health issues. In the United States, 61% of adults search online and 39% use social media such as Facebook for health information [17]. An empirical research found that Twitter and Facebook can engage citizens in two-way communications [18]. When focusing on social media for health communication, it is important to understand of these technologies, such as Facebook, Twitter, and YouTube, their impact on health communication. Governments provide correct health information through websites that not only increase the visibility of governance, but also give the public an effective method for obtaining information and to achieve interactive exchanges that improve public participation and increase knowledge and literacy in various areas [14, 19].

“Long-term care” isn’t only the government’s most promoted health policy, it also receives the most public response. This result shows concerns about Taiwan’s aging population and the growing number of elderly people in society because both government officials and the public care deeply about this issue. Before the MoHW was founded, long-term care policies were jointly set by the Ministry of the Interior, the Council for Economic Planning and Development, and the Health Administration with the Ministry of the Interior’s Social Affairs Administration in an attempt to provide broad solutions [16]. After the MoHW was founded, long-term care-related operations were transferred to the “Department of Nursing and Health Care,” as supported by the data results of this study. Also, the requirements for long-term care have become a major health and welfare issue as the population continues to age and the number of feeble elderly increases. Since 1996, the government has continued to plan projects to address this problem, primarily basing solutions in 2007’s “10 Year Long-term Care Plan,” 2015’s “Long-term Care Service Act,” and 2016’s “10 Year Long-term Care Plan 2.0.” These solutions originally serviced elderly individuals above the age of 65, indigenous people between the ages of 55 and 64, those with mental disabilities between the ages of 50 and 64, and elderly individuals living alone who are IADLs feeble. However, it was expanded to include individuals above 50 with slight mental disabilities, those under 50 with disabilities, the feeble elderly above age 65, and indigenous people with disabilities between the ages of 55 and 64 to address the growing population of mentally or physically disabled people and the demand for long-term care [15]. However, everyone experiences “birth, aging, disease, and death,” so anyone has the opportunity to use long-term care services. Long-term care services not only cater to the service target, but also provide a necessary break for relatives and care givers. In other words, this massive and complicated issue and its related services cannot be explained to the public with a few simple sentences. This phenomenon also relates to the results of this study. While the Department of Nursing and Health Care doesn’t have the largest number of posts, it received the largest amount of public response, and the MoHW’s 2017 Annual Project Plan showed policies and posts focused on long-term care that also received the highest public response. This shows that both government and the public care more about long-term care issues compared to other policies.

The results of this study show that aside from long-term care, the public also cares deeply about issues such as medical care and food- and drug-related incidents. Medical incidents in 2017 included problems with emergency medical protocols due to flu, condemning improper coverage disparaging the image of nursing, using World Hospice and Palliative Care Day to highlight DNRs, using seminars with celebrities to teach the public about organ donation, and promoting medical services for indigenous people/remote areas. Food- and drug-related incidents at the time included an incident involving fake CRESTOR medication on the market, identifying Chinese medicine ingredients or inappropriate transaction behavior, control of expired drugs, children’s abuse of drugs, improper food labeling, the allowing of beef imports, regulations on creamer ingredients and labeling, among others. It can be seen that the MoHW is highly effective in policy announcements and that it uses internet resources to provide the public with correct information in regard to health and welfare. Articles provided by Taiwan’s government not only address issues of public scrutiny, but also provide information that can often be neglected or unknown to greatly increase public awareness and their understanding and recognition of health and welfare issues.

Of the agencies, SFAA and HPA primarily use Facebook to advocate policy, mainly promoting content related to long-term care, welfare services for minority groups, and physical and mental health promotion. These two major agencies are more adept at using internet media to market policies and increase public absorption of related knowledge that help achieve goals in governance. Also, the results of this study show that the greatest number of agencies are involved in policy related to “physical and mental health promotion.” This phenomenon now encompasses a broader scope compared to other policies in the 2017 annual project plan and has received support such as the Department of Mental and Oral Health and their networks in anti-drug use for children, suicide prevention, and mental health; HPA provides help to quit smoking, internet addiction, or osteoporosis. SFAA has child services (birth registration, health insurance for newborns, etc.). CDC advocate clearing disease sources. The Department of Chinese Medicine and Pharmacy discusses issues of supplementary foods. The Department of Planning’s release of quarterly flyers show the results of participation through multiple agencies. Looking at other policies with a more uniform nature or targeted towards specific groups, the number of agencies that can participate is severely limited. For example, prevention of infectious disease and epidemics and response policies is mainly the responsibility of CDC while FDA primarily manages food and drug policy.

This study primarily uses NodeXL to analyze the MoHW network relationships to explore their promotions and degree of interaction with the public. This analysis benefited MoHW agencies and helped them reflect on policies with insufficient advocacy. For example: in Fig. 1, the budget allocation policy does not appear in the network relationship figure, so in other words, the fan page made no mention of related information or articles at the time. Furthermore, while the cause and effect relationship of the public and postings cannot be confirmed, meaning that public attention on issues can occur before or after a post, but the issues that the public care about can be identified. For example, Fig. 4 shows that long-term care, medical care, and food and drug policies receive the highest response rate, which is beneficial for future consideration of agencies in promoting policies. This study found that some agencies lack advocacy on the MoHW fan page, but this might be due to them having their own fan pages, where they focus their marketing efforts such as CDC’s “1992 Epidemic Prevention Master.”

In addition, although commenting on posts may not be because a policy is more controversial. Also it is important not to see any necessary relationship between the number of comments on a post and the number of reads and indeed interest. Facebook user activities including like, comment, share are related to posting time as well as general interests of public at that time/period, most studies have investigated Facebook page to understand the user thought and how effectiveness and efficiency for the post [20–24]. Thus, we consider that each comment on the post either positive or negative response regarded as which post were attracted the attention of the public compared the public who did not response, so this quantitative study shows the relatively potential interactions between agencies, policies, and the interest of the public.

## Conclusions

Current use of internet media to advocate policy to interact with the public has become emerging strategies. While the research cannot extrapolate the promotional results in other years, this study’s social network analysis of Taiwan’s top level government agency fan pages divulges the government’s most emphasized core policies and the strength of the correlations between agencies and policies, especially in terms of intergovernmental cooperation related to policies. In addition, the degree of public response can also be relatively obtained. Further exploring the characteristics of these publics in the future may particular useful for successful marketing strategy.

## Supplementary information

**Additional file 1.** MoHW administrative organization chart (2017).

## Data Availability

All data analyzed in this study were from the open data on the Facebook fan page.

## Abbreviations

MoHW: the Ministry of Health and Welfare
NodeXL: Network Overview, Discovery and Exploration for Excel
CDC: Centers for Disease Control
FDA: Food and Drug Administration
NHA: National Health Insurance Administration
HPA: Health Promotion Administration
SFAA: Social and Family Affairs Administration
NPSC: National Pension Supervisory Committee
