# Supplementary figures and images for: Understanding public interest and needs in health policies through the application of social network analysis on a governmental Facebook fan page
Source: BMC Public Health. 2020 Sep 7;20:1367. doi: 10.1186/s12889-020-09420-y (PMC7487966; doi:10.1186/s12889-020-09420-y)

# MoHW administrative organization chart (2017)

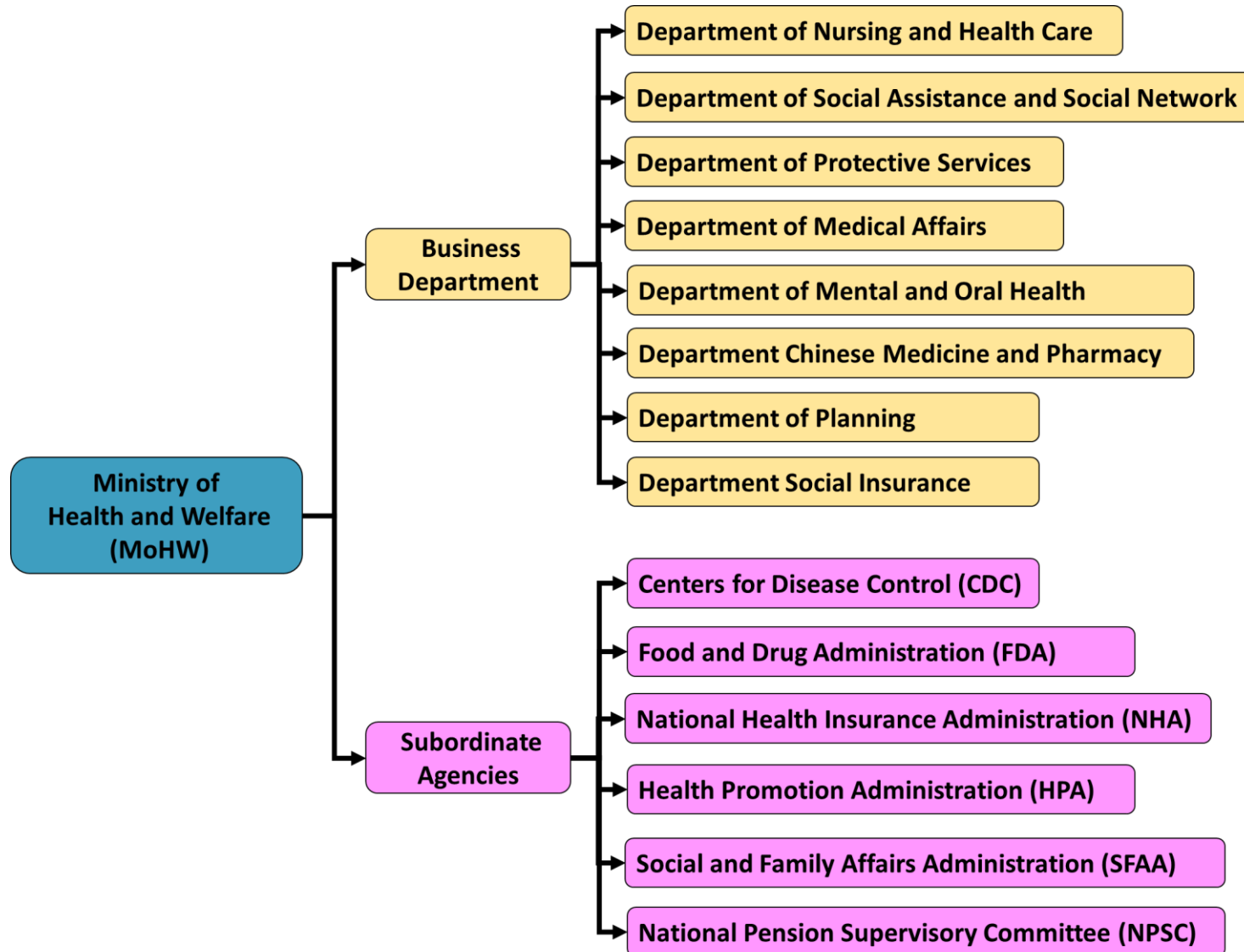

Supplement: Supplementary file 1 — Additional file 1. MoHW administrative organization chart (2017). [file 12889_2020_9420_MOESM1_ESM.pdf]
